# HIV-associated cavernous sinus disease

**DOI:** 10.4102/sajhivmed.v20i1.862

**Published:** 2019-03-20

**Authors:** Cait-lynn D. Wells, Anand A. Moodley

**Affiliations:** 1 Department of Neurology, Greys Hospital, University of KwaZulu-Natal, Pietermaritzburg, South Africa; 2 Department of Neurology, Universitas Hospital, University of the Free State, Bloemfontein, South Africa

## Abstract

**Introduction:**

The underlying diagnosis of cavernous sinus disease is difficult to confirm in HIV-coinfected patients owing to the lack of histological confirmation. In this retrospective case series, we highlight the challenges in confirming the diagnosis and managing these patients.

**Results:**

The clinical, laboratory and radiological data of 23 HIV-infected patients with cavernous sinus disease were analysed. The mean age of patients was 38 years. The mean CD4+ count was 390 cells/μL. Clinically, patients presented with unilateral disease (65%), headache (48%), diplopia (30%) and blurred vision (30%). Third (65%) and sixth (57%) nerve palsies in isolation and combination (39%) were most common. Isolated fourth nerve palsy did not occur. Tuberculosis (17%) was the most commonly identified disorder followed by high-grade B-cell lymphoma (13%), meningioma (13%), metastatic carcinoma (13%) and neurosyphilis (7%). In 22% of the patients, there was no confirmatory evidence for a diagnosis. The patients were either treated empirically for tuberculosis or improved spontaneously when antiretroviral therapy was started. Cerebrospinal fluid was helpful in 4/13 (31%) of patients where it was not contraindicated. Only 3/23 (13%) of the patients had a biopsy of the cavernous sinus mass. The outcomes varied, and follow-up was lacking in the majority of patients.

**Conclusion:**

In HIV-infected patients, histological confirmation of cavernous sinus pathology is not readily available for various reasons. In resource-limited settings, one should first actively search for extracranial evidence of tuberculosis, lymphoma, syphilis and primary malignancy and manage appropriately. Only if such evidence is lacking should a referral for biopsy be considered.

## Introduction

The cavernous sinus, a venous structure at the base of the skull, contains important neurological and vascular components that are susceptible to opportunistic infections, para-infectious disorders and neoplastic disorders in HIV-infected patients. The cavernous sinuses are two dura-enclosed venous chambers connected by the circular sinus.^1^ The crossover of pathology between the two sides is therefore not uncommon. Each cavernous sinus receives venous blood from the superior and inferior ophthalmic veins and drains via the superior and inferior petrosal sinuses into sigmoid sinuses bilaterally. The involvement of vital structures within the cavernous sinus presents as a double-edged sword. They allow for early detection of cavernous sinus disease, but their presence also heralds the presence of grave pathology.

**FIGURE 1:**
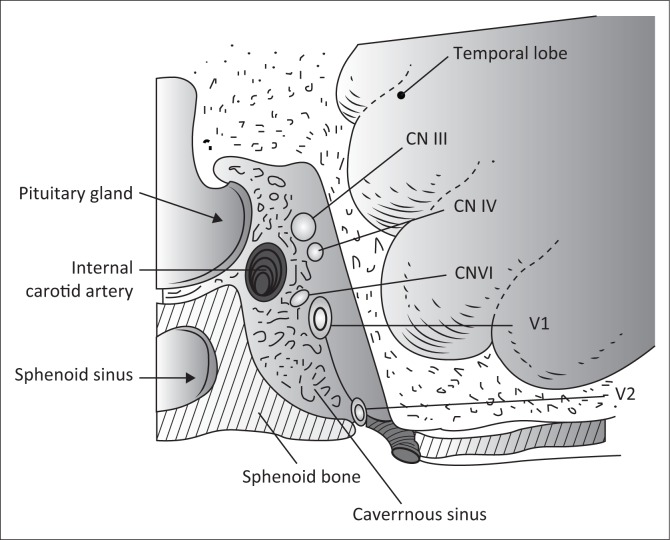
The cavernous sinus showing cranial nerves III, IV, ophthalmic division of V (V1) and maxillary division of V (V2) along the lateral wall of the sinus. The VI cranial nerve lies free within the sinus. The sympathetic fibres, which enter the cavernous sinus along the carotid artery, are not shown.

Each cavernous sinus contains the carotid artery and the sixth cranial nerve lying within the sinus (Figure 1). Sympathetic nerves that emerge from the carotid artery wall run along the sixth nerve for a short distance and are then destined for the eye along the nasociliary branch of the fifth cranial nerve. From rostral to caudal, the third, fourth and ophthalmic divisions of the fifth cranial nerve lie within the lateral wall of the sinus and further back is a short encounter with the maxillary division of the fifth nerve, which enters via the foramen rotundum en route to the Gasserian ganglion. The cavernous sinus syndrome is defined as involvement of two or more of the third, fourth, fifth and sixth cranial nerves or involvement of any amount of cranial nerves with neuro-imaging confirming the presence of a cavernous sinus lesion. Clinically, various combinations of third nerve, fourth nerve, sixth nerve, Horner syndrome, ophthalmic and maxillary division sensory loss are localised to the cavernous sinus. The cavernous sinus is also secondarily affected by pathology in surrounding structures, namely, the pituitary gland, the surrounding dura, the optic chiasm, the sphenoid sinus and structures of the floor of the third ventricle. Lesions of the cavernous sinus that spread anteriorly to the orbital apex affect the optic nerve.^2^

Causes of cavernous sinus pathology are protean. In a series of 151 patients, Keane et al. described the common causes for cavernous sinus lesions to be tumours (30%), trauma (24%) and self-limiting inflammation (23%), while carotid aneurysms, carotico-cavernous fistulae, infection and other causes constituted the remaining 12%. The most common tumour in this series was nasopharyngeal carcinoma (22%), followed by metastases (18%) and lymphoma (18%). The infectious aetiologies comprised one case of bacterial sphenoid sinusitis and three cases of mucormycosis. In this series, there were three patients who were HIV-infected, and all three were diagnosed with lymphoma.^3^

Fernandez et al. in their series of 126 patients found the most common cause for cavernous sinus disease to be tumours (63%), vascular lesions (20%), inflammatory conditions such as Tolosa-Hunt syndrome (13%) and miscellaneous causes (4%). Of the miscellaneous causes, only two were because of infectious aetiologies, namely, aspergillosis and thrombophlebitis from *Haemophilus influenzae* infection. Pituitary adenoma and meningioma were the most frequent of the tumours at 36.25% and 31.25%, respectively. In this series, two HIV-infected patients formed part of the cohort – both patients were diagnosed with lymphoma.^4^

In the HIV population, one would predict that the disease spectrum would coincide with the immunocompromised state of the patient, and opportunistic infections and HIV-associated neoplastic disorders would be commonly encountered. However, epidemiological data regarding cavernous sinus pathology in HIV-infected patients are lacking. The deep skull base location of the cavernous sinus and the fact that most patients are severely immunosuppressed discourage invasive diagnostic procedures unless lesions are easily accessible via the paranasal sinuses. Histological confirmation is therefore scant. Often, the diagnosis is based on the presence of a systemic disease, or patients are treated empirically for the commonly occurring diseases. The outcome therefore can be unpredictable and catastrophic at times.

There is no study that has specifically addressed cavernous sinus pathology in patients coinfected with HIV. The literature in this population consists of mainly case reports (Table 1). Surprisingly, tuberculosis (TB) of the cavernous sinus has not been described in HIV-infected patients previously. In fact, TB of the cavernous sinus is an uncommon disorder even in an immunocompetent patient.^5^ The only infection reported is aspergillosis. Infection as an infrequent cause of cavernous sinus disease in HIV-infected patients is likely because of poor reporting or lack of confirmatory evidence.

**TABLE 1:** Case reports of patients infected with HIV and presenting with cavernous sinus disease.

Author (year)	Relevant data	Diagnosis
Gross et al.^12^	35-year-old female	Eosinophilic granuloma
Keane^3^	3 patients	B-cell lymphoma
Kleinschmidt-Demaster et al.^13^	35-year-old, unilateral mass	Leiomyosarcoma
Blumenthal et al.^14^	43-year-old male, CD4 23 cells/μL	Leiomyosarcoma
Fernandez et al.^4^	2 patients	B-cell lymphoma
Dhillon and Shah ^15^	25-year-old CD4 218 cells/μL	B-cell lymphoma
Junior et al.^16^	51-year-old with bilateral lesions	Non-Hodgkin’s lymphoma
Meltzer et al.^17^	53-year-old with nasopharyngeal mass	Nasopharyngeal carcinoma
Humphrey et al.^18^	47-year-old female, CD4 214 cells/μL	Aspergillosis

We undertook a retrospective analysis to highlight the pathology and challenges encountered in the diagnosis and management of HIV-infected patients with cavernous sinus disease. Non-HIV-infected patients have similar challenges, but despite equity in the management of HIV-infected patients, personal experience shows us that surgeons are more likely to offer biopsies to non-HIV-infected patients for the fear of negative outcomes post-surgery in HIV-infected patients.

Twenty-three HIV-infected patients with cavernous sinus lesions as their main reason for referral were recruited. Their clinical, radiological and biochemical data were analysed. Problems encountered in their diagnosis and management will be highlighted.

## Method

All HIV-infected patients, from 2010 to 2016, referred to the neurology department of Grey’s Hospital, Pietermaritzburg, with non-traumatic cavernous sinus lesions as their main presenting problem were included for analysis. The data search was done by selecting the keywords of cavernous sinus and HIV in hospital records and patient summaries. Clinical, biochemical and radiological data were obtained from hospital records. All data were captured on a spreadsheet for descriptive statistics. Averages and standard deviations were obtained for continuous quantitative data and qualitative data were represented in tables and graphs.

## Ethical consideration

Ethical approval (BE370/15) for a retrospective analysis was obtained from the University of KwaZulu-Natal biomedical research ethics committee.

## Results

A total of 23 HIV-infected patients with cavernous sinus disease were recruited for analysis. The mean age of the patients was 38 years (Range 22–62 years), and 52% (12/23) were female. Eight patients were newly diagnosed with HIV infection. The remainder of the patients were aware of their HIV infection for 1–6 years. The mean CD4+ count was 390 cells/μL ± 227 (s.d.), implying mild-to-moderate immunosuppression. However, two patients had severe immunosuppression with CD4+ counts of 24 cells/μL and 70 cells/μL, respectively. The viral load was not readily obtained owing to inadequate records. Four patients had undetectable viral loads. In three patients, viral loads of 54900 copies/mL, 850 copies/mL and 4992 copies/mL were obtained. Viral loads for the remaining patients were unknown.

**FIGURE 2:**
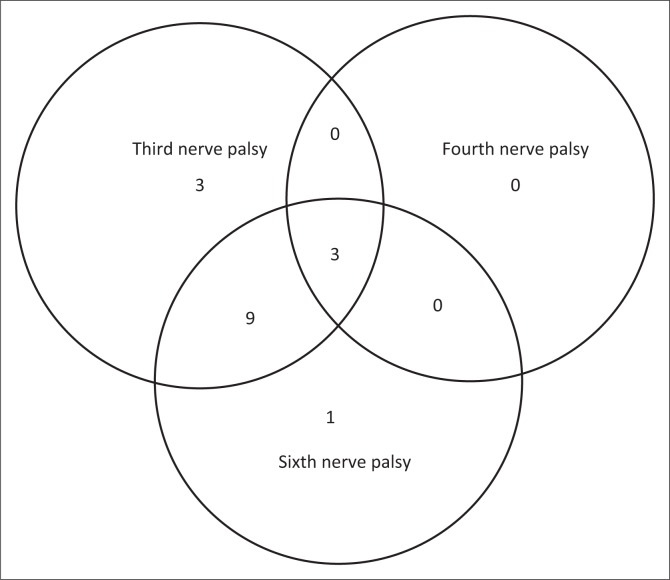
Number of patients presenting with various combinations of third, fourth and sixth cranial nerve palsies.

Acute onset of headache was common, but visual symptoms of diplopia and blurred vision occurred in only 30% of the patients (Table 2). Proptosis was an uncommon finding, and unilateral disease was present in 65% (15/23) of patients. Isolated third nerve and sixth nerve palsies were common (65% and 57%, respectively; Figure 2). Fifty-two percent (12/23) of the patients had a combination of third, sixth and fourth palsies or third and sixth nerve palsies. Isolated fourth nerve palsy did not occur and when present only occurred with combined third and sixth nerve palsy. The involvement of the fifth cranial nerve was found only in 22% (5/23) of the patients. Systemic clinical findings occurred in 57% (13/23) of the patients but only contributed to the diagnosis in 35% (8/23) of the patients.

**TABLE 2:** Symptoms, signs and associated findings.

Variable	Number (23)	Percentage
**Symptoms**		
Headache	11	48
Peri-orbital pain	3	13
Diplopia	7	30
Droopy eyelid	8	35
Blurred vision	7	30
Facial weakness and/drooling	4	17
Deafness	1	4
**Signs**		
Proptosis	1	4
Unilateral disease	15	65
Bilateral disease	8	35
Third nerve palsy	15	65
Fourth nerve palsy	3	13
Sixth nerve palsy	13	57
Third and fourth nerve palsies	0	0
Third and sixth nerve palsies	9	39
Fourth and sixth nerve palsies	0	0
Third, fourth and sixth nerve palsies	3	13
Ophthalmic division of fifth nerve	5	22
Maxillary division of fifth nerve	1	4
Horner syndrome	2	9
Decreased visual acuity	7	30
Abnormal fundoscopy	4	17
**Associated findings**		
Seventh nerve palsy	4	17
Generalised lymphadenopathy	2	9
Hemiplegia	1	4
Pulmonary TB	2	9
Breast carcinoma	2	9
Nasopharyngeal carcinoma	1	4
Ocular toxoplasmosis	1	4

TB, tuberculosis.

**FIGURE 3:**
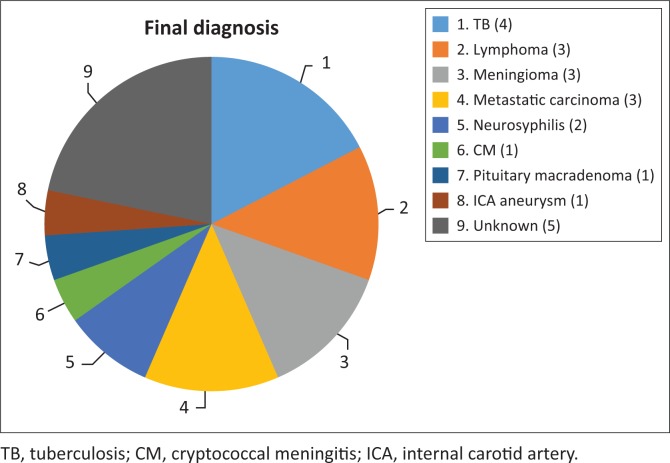
Pie chart showing the number of patients and the spectrum of cavernous sinus disease in HIV-infected patients.

Figure 3 depicts various diagnoses made, and Table 3 shows the radiological features of the 18 patients. In five cases, the diagnosis was unknown owing to the absence of clinical, biochemical or radiological evidence for the underlying pathology. The most commonly identified diagnosis was TB. Tuberculosis was confirmed on cerebrospinal fluid (CSF) in one patient. The other three patients had positive sputum culture for TB or suggestive chest X-ray and responded neurologically to anti-TB treatment alone making TB of the cavernous sinus highly probable. High-grade B-cell lymphoma, metastatic carcinoma and meningioma were the next most common.

**TABLE 3:** Radiological features of 18 patients with confirmed diagnosis (the diagnosis was unknown in five patients).

Confirmed diagnosis (number)	Imaging of representative cases	Description of imaging findings	Diagnostic confirmation
Tuberculosis (4)	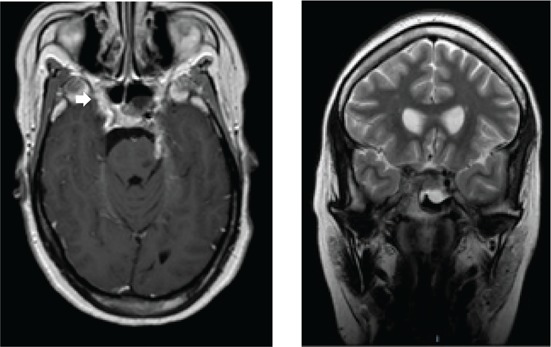	4 – bilateral abnormal CS enhancement (arrow) 3 – associated meningeal enhancement	2 – known with pulmonary TB 1 – CSF suggestive of TB 1 – CXR suggestive of TB (sputum negative)
High-grade B-cell lymphoma (3)	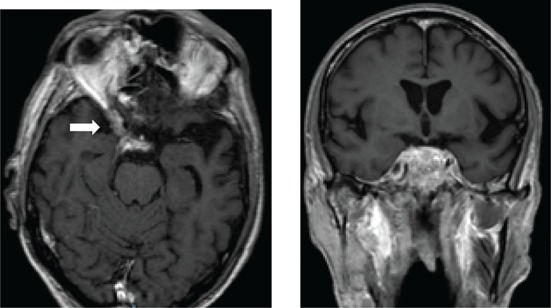	2 – unilateral CS enhancement (arrow) 1 – Bilateral CS enhancement 1 – orbital extension	2 – Lymph node biopsy 1 – Biopsy of lung mass
Meningioma (3)	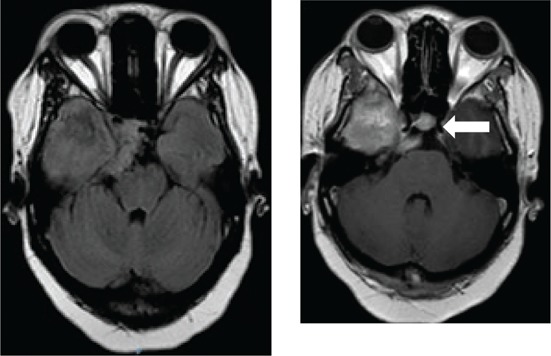	3 – unilateral CS lesion 1 – Unilateral CS lesion with optic nerve involvement (arrow) 1 – encasement of Internal carotid artery	2 – biopsy of lesion 1 – Based on radiological features
Metastases or local invasion (3)	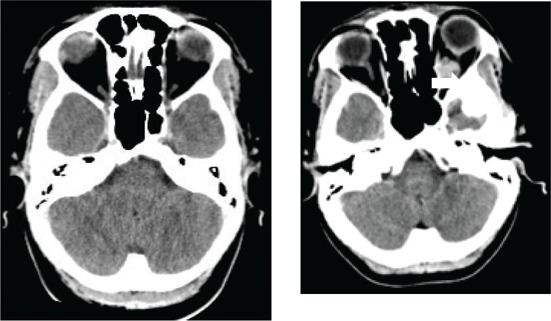	1 – Unilateral CS mass with orbital extension(arrow) 1 – Multiple metastases. CS syndrome but no lesion noted 1 – local invasion of nasopharyngeal carcinoma	1 – Known breast carcinoma with lung metastases 1 – Corneal mass biopsy (squamous cell carcinoma) 1 – Biopsy proven nasopharyngeal carcinoma
Neurosyphilis (2)	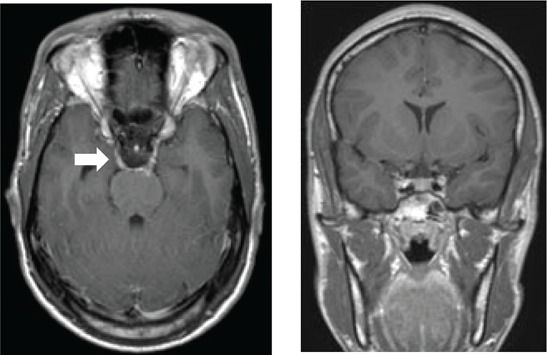	1 – enhancement of abducens nerves within CS (arrow) 1 – Bilateral CS enhancement	Both patients had RPR titre >1:32 and abnormal CSF pleocytosis
Cryptococcal meningitis (1)	No images available	Unilateral CS mass	Positive CSF cryptococcal antigen test
Pituitary adenoma (1)	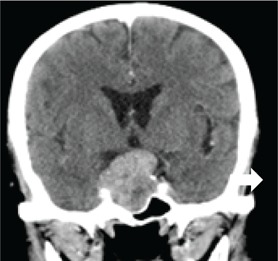	Pituitary macroadenoma with invasion into the CS (arrow)	Histology confirmed pituitary macroadenoma with immunopositivity to LH and Prolactin
Internal carotid artery aneurysm (1)	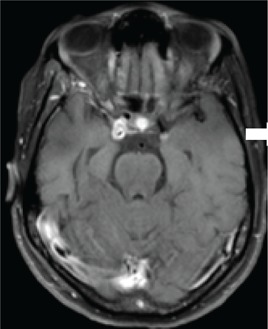	Right internal carotid artery saccular aneurysm with wall enhancement (*arrow*)	Confirmed on CT angiogram

CS, cavernous sinus; TB, tuberculosis; CT, computerised tomography; CSF, cerebrospinal fluid; CXR, chest X-Ray; RPR, rapid plasma regain; LH, Luteinising hormone.

Table 3 shows 18 patients (78%) with confirmed diagnosis. Representative images of the common groups are shown. In five patients (22%) where no diagnosis was confidently made, two patients were treated empirically for TB and were lost to follow-up after discharge; one patient was treated for pyogenic sinusitis and the patient’s condition subsequently improved; one patient who had acute inflammatory demyelinating polyneuropathy (AIDP) and was treated with intravenous immunoglobulin, recovered and was discharged. He had abnormal cavernous sinus enhancement; so, his eye signs were not attributed to an AIDP variant such as Miller Fisher syndrome. The fifth patient had a cavernous sinus syndrome that cleared after the commencement of antiretroviral therapy. We presume the latter two to have been para-infectious in nature.

CSF was obtained in 13 patients. It was contraindicated or not required in the others. In 31% (4/13) of the cases, the CSF was abnormal and pointed to the diagnosis. In only 25% (1/4) of the cases, TB was confirmed on CSF findings. Abnormal CSF findings confirmed the diagnosis of cryptococcal meningitis in one patient and neurosyphilis in two patients. All three patients were treated appropriately but none returned for follow-up CSF examination. Neurosyphilis was confirmed in two patients by serum rapid plasma regain titres of > 1:32. CSF pleocytosis and the positive venereal disease research laboratory tests were confirmatory in both cases.

The case of cryptococcal meningitis with pachymeningitis and unilateral cavernous sinus syndrome occurred in a patient with CD4+ count of 338 cells/μL. The CSF was mildly pleocytotic (polymorphs = 3 cells/μL and lymphocytes = 113 cells/ μL), protein was 0.81 g/L and glucose was 3.1 mmol/L. Diagnosis was made on a positive cryptococcal antigen test. Response to amphotericin B and fluconazole was initially promising, but follow-up was absent.

The outcomes varied, and the follow-up of patients was lacking. Two patients with confirmed TB returned for follow-up and were recovering on treatment. The three patients with high-grade B-cell lymphoma were referred to oncology and lost to follow-up. The three patients with meningioma were referred to neurosurgery for further follow-up. Two patients have since received radiotherapy but remain clinically unchanged. The patient with breast carcinoma demised from pulmonary embolism. The patients with nasopharyngeal carcinoma and corneal carcinoma were receiving chemotherapy from oncology, but follow-up showed no change to the cavernous sinus syndrome. The patient with the pituitary macroadenoma had debulking surgery and was commenced on replacement therapy by endocrinology. Her visual acuity loss and left-sided ophthalmoplegia have not improved; she is awaiting radiotherapy. The patient with the cavernous sinus aneurysm was still awaiting neurosurgical intervention three months after diagnosis.

## Discussion

According to 2016 statistics, South Africa has the most high-profile HIV epidemic in the world, having 7.1 million people living with the infection.^6^ Despite having the largest antiretroviral treatment programme globally, the complications from HIV infection continue to burden the limited health resources in South Africa. Determining the actual duration of HIV infection is difficult for various reasons, which include late presentation and reluctance to know one’s HIV status for the fear of stigmatisation and apathy. Despite regular and widely published educational, screening and treatment programmes for HIV infection in South Africa, stigmatisation of the illness is still prevalent.^7^

Neurological sequelae of HIV infection are common, and cavernous sinus disease in the setting of HIV infection poses huge challenges both for diagnosis and management. Cavernous sinus disease biopsies by neurosurgery even in a tertiary facility are at most times unobtainable. Trauma consumes most of their time leaving little to disorders where empirical treatment is the fall-back option. Histological diagnosis while indicated becomes unobtainable and if systemic evidence is unavailable then confirmation of the diagnosis is extremely difficult to obtain. In the setting of cavernous sinus disease, even empirical treatment is unsupported as epidemiological data regarding cavernous sinus disease are unavailable in South Africa. The different spectrum of disease in developed countries cannot be extrapolated to developing countries. So, management strategies usually fall back on expert opinion. Furthermore, despite the excellent HIV education and antiretroviral treatment programme in South Africa, non-adherence to treatment by patients is common.^8^ The follow-up of patients is inconsistent despite the high cost of investigations and treatment initially given to patients at the tertiary facilities.

In this study, we have attempted to obtain reliable and perhaps reproducible epidemiological data on cavernous sinus disease in HIV-infected patients but, as expected, were faced with many challenges. Of the 23 patients with cavernous sinus disease, the diagnosis was confidently made in 18 patients (78%). The majority had mild-to-moderate immunosuppression. Only two patients had severe immunosuppression of CD4+ < 100 cells/μL. Histological diagnoses from the biopsy of the cavernous sinus disease was obtained in 13% (3/23) of the patients. Systemic evidence and/or expert opinion on radiological findings were used for the other cases. In five patients, the cause was unknown. Fortunately, two patients recovered without specific intervention, and one responded to intravenous antibiotics. Two patients were treated empirically for TB based solely on radiological findings as their CSF findings were unhelpful. Both patients did not return for follow-up, and the outcome of their treatment remains unknown.

The most commonly identified diagnosis was TB with evidence available from pulmonary TB in all four patients. CSF results were only contributory in one patient. High-grade B-cell lymphoma, metastatic carcinoma and meningioma were the next most common. With immunosuppression, the presence of high-grade B-cell lymphoma was plausible, but non-HIV-related metastatic disease and meningioma were probably incidental.

Two cases of neurosyphilis were identified in this series, which is high as there are only two other cases in the literature of neurosyphilis affecting the cavernous sinus where HIV was negative or the HIV status was unknown.^9,10^ Neurosyphilis is more common in HIV-infected patients, which could account for the higher number found in this series affecting an atypical site. Response to treatment was much too early to assess during admission and unfortunately both patients failed to return for follow-up. There were isolated cases of a saccular internal carotid artery aneurysm and a pituitary macroadenoma, which were also probably incidental. The increased incidence of intracranial saccular aneurysms in HIV infection is unsubstantiated; however, dolichoectatic vessels from immune-mediated vessel damage are more plausible.^11^ In this first case series of cavernous sinus disease in HIV-coinfected patients, we describe HIV- and non-HIV-related pathology.

The third cranial nerve was the commonest cranial nerve affected in this group followed by involvement of the sixth cranial nerve, the ophthalmic division of the fifth cranial nerve and then the fourth cranial nerve. Unlike the third and sixth cranial nerve palsies, the fourth nerve palsy did not occur in isolation. The Horner syndrome was present in one patient only. This could imply a rare occurrence or difficulty in detection in the presence of other cranial nerve palsies. Unilateral disease was present in 65% of the patients despite the connection of the two cavernous sinuses by the circular sinus. Proptosis and visual impairment were uncommon implying minimal extension of pathology from the cavernous sinus to the orbital apex.

The magnetic resonance imaging and computed tomography findings of cavernous sinus disease were helpful in localising the disorder but not in elucidating the pathology in the majority of cases. The presence of unilateral or bilateral enhancing masses did not provide any clues to the underlying pathology. However, MRI was diagnostic in the case of the pituitary adenoma and saccular internal carotid artery disease, which, in all probability, were incidental disorders.

CSF findings were positive in confirming the diagnosis in 31% (4/13) of the patients, which included one patient with TB, one patient with cryptococcal meningitis and two patients with neurosyphilis. While treatment at the tertiary centre was appropriately initiated, continuation and follow-up care was poor. It is not unusual for patients to ‘disappear’ into a void or be lost in the system after discharge from the tertiary centre. In most instances, the fault lies with the highly prohibitive referral system to tertiary and academic centres in South Africa. The apathy and lack of social support are other contributing factors.

This study was a retrospective chart review and hence fraught with many limitations as evidenced by a deficiency of appropriate and detailed record-keeping. Furthermore, the follow-up of patients was poor, especially when referred to other departments for co-management.

**FIGURE 4:**
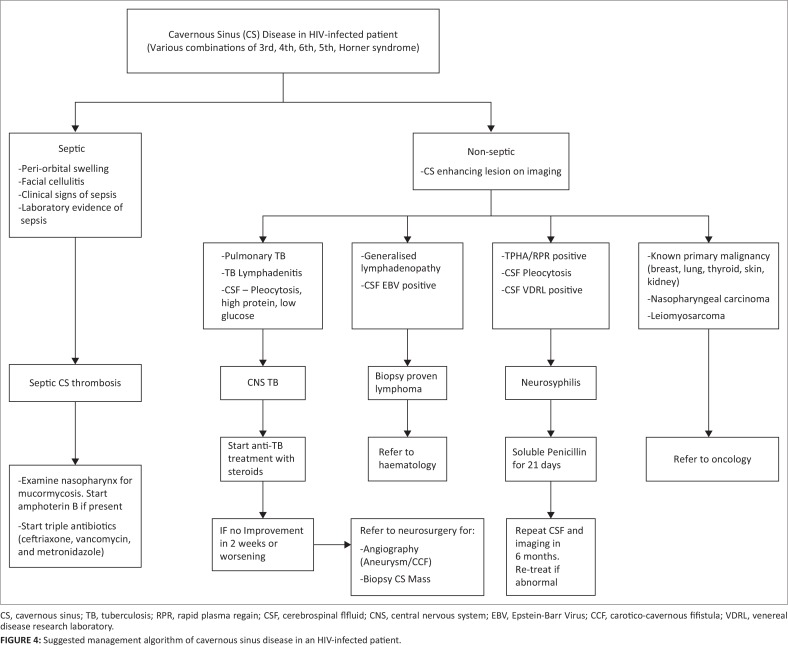
Suggested management algorithm of cavernous sinus disease in an HIV-infected patient.

In resource-limited developing countries, access to tertiary care is a challenge. Neurosurgical services are usually inaccessible, but a rational approach to diagnosis and treatment is achievable. We suggest a management strategy for HIV-infected patients presenting with cavernous sinus disease that can address the common causes at regional level before referral to a tertiary centre (Figure 4).

There is a growing need for comprehensive databanks in South Africa for epidemiological research and the benefits thereof. In addition, HIV infection as a serious but manageable disorder needs to be revisited and a re-emergence of a rigorous HIV education programme is essential for both the general public and health care workers alike. Cavernous sinus disorders, as with many other neurological complications, are preventable. Negligence in the preservation of a patient’s immunity can be catastrophic, especially in a disorder that is not easy to diagnose or manage.
